# Unexpected Gating Behaviour of an Engineered Potassium Channel Kir

**DOI:** 10.3389/fmolb.2021.691901

**Published:** 2021-06-10

**Authors:** Charline Fagnen, Ludovic Bannwarth, Dania Zuniga, Iman Oubella, Rita De Zorzi, Eric Forest, Rosa Scala, Samuel Guilbault, Saïd Bendahhou, David Perahia, Catherine Vénien-Bryan

**Affiliations:** ^1^ UMR 7590, CNRS, Muséum National d’Histoire Naturelle, Institut de Minéralogie, Physique des Matériaux et Cosmochimie, IMPMC, Sorbonne Université, Paris, France; ^2^ Laboratoire de Biologie et de Pharmacologie Appliquée, Ecole Normale Supérieure Paris-Saclay, Centre National de la Recherche Scientifique, Gif-sur-Yvette, France; ^3^ Department of Chemical and Pharmaceutical Sciences, University of Trieste, Trieste, Italy; ^4^ IBS University Grenoble Alpes, CNRS, CEA, Grenoble, France; ^5^ Faculté de Médecine, CNRS UMR7370, LP2M, Labex ICST, University Côte d’Azur, Nice, France

**Keywords:** molecular dynamics and normal modes, HDX-mass spectrometry, single channel recording, potassium channel KirBac3.1, mutation effect

## Abstract

In this study, we investigated the dynamics and functional characteristics of the KirBac3.1 S129R, a mutated bacterial potassium channel for which the inner pore-lining helix (TM2) was engineered so that the bundle crossing is trapped in an open conformation. The structure of this channel has been previously determined at high atomic resolution. We explored the dynamical characteristics of this open state channel using an *in silico* method MDeNM that combines molecular dynamics simulations and normal modes. We captured the global and local motions at the mutation level and compared these data with HDX-MS experiments. MDeNM provided also an estimation of the probability of the different opening states that are in agreement with our electrophysiological experiments. In the S129R mutant, the Arg129 mutation releases the two constriction points in the channel that existed in the wild type but interestingly creates another restriction point.

## Introduction

A detailed study of function requires careful dissection of the mechanistic steps. Protein engineering can provide a powerful tool for studying the relationships between structure and function. The design of various potassium channels with carefully chosen replacement residues has helped describe the gating mechanism of these channels. For instance, we can mention mutations close to the selectivity filter (Capener et al., 2003), on the wall of the cytoplasmic pore (Fujiwara and Kubo, 2006), on the cytoplasmic domain (Inanobe et al., 2013), at the end of the cytoplasmic pore (Pegan et al., 2005), at the extracellular domain of Kir2.2 (Li et al., 2014), at the bottom of the bundle crossing (Linder et al., 2015), or at the level of the cytoplasmic domain subunit interfaces (Wang et al., 2017). All these investigations, either *in silico* or experimental (NMR, FRET, etc.) provided valuable information.

**FIGURE 1:**
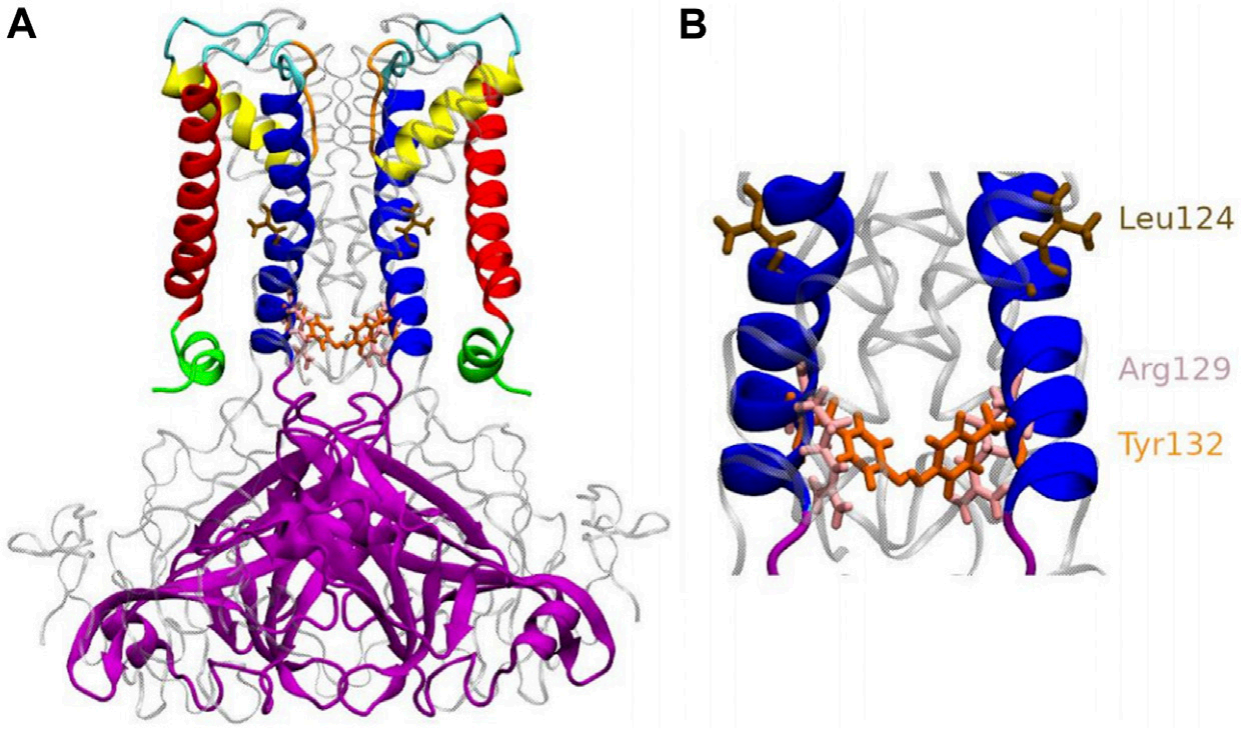
Anatomy of KirBac3.1 S129R from the modeled KirBac3.1 S129R **(A)** The transmembrane portion of each monomer of KirBac3.1 is composed of four helices: slide helix (green), transmembrane helix 1 (red), pore helix (yellow), and transmembrane helix 2 (blue). The mutation S129R is situated at the level of the helix bundle (pink), at the bottom of the inner helix **(B)** Leu124 and Tyr132 are shown in color brown and orange respectively. Arg129 is shown in pink.

A few years ago, the open state kir channel’s crystal structure was revealed by KirBac3.1 S129R (Bavro et al., 2012; Zubcevic et al., 2014), which was designed so that the channel was trapped in an open conformation. Indeed, before the publication of this structure, most structures were known in the closed state, with the conduction pathway occluded. The use of an engineered protein made it possible to observe for the first time at high resolution the KirBac channel with the bundle-crossing gate in an open conformation, where the constriction points (Leu124 and Tyr132) are released (Bavro et al., 2012) as shown in Figure 1. This structure allowed proposing a mechanism for opening the channel. In this structure, we noticed that the mutated residue Arg was facing the channel’s center and therefore could create the condition for another unexpected constriction point. However, this channel is functionally open (Paynter et al., 2010) and we did not notice any toxic effect of this engineered protein on the host cell (De Zorzi et al., 2013). We then decided to investigate further this mutated channel’s function and dynamics using an experimental and *in silico* study, allowing us to explain its particular behavior, which, despite unexpected characteristics, provided valuable information on the open state structure.

## Results and Discussion

### Hydrogen/Deuterium Exchange Coupled to Mass Spectrometry

**FIGURE 2:**
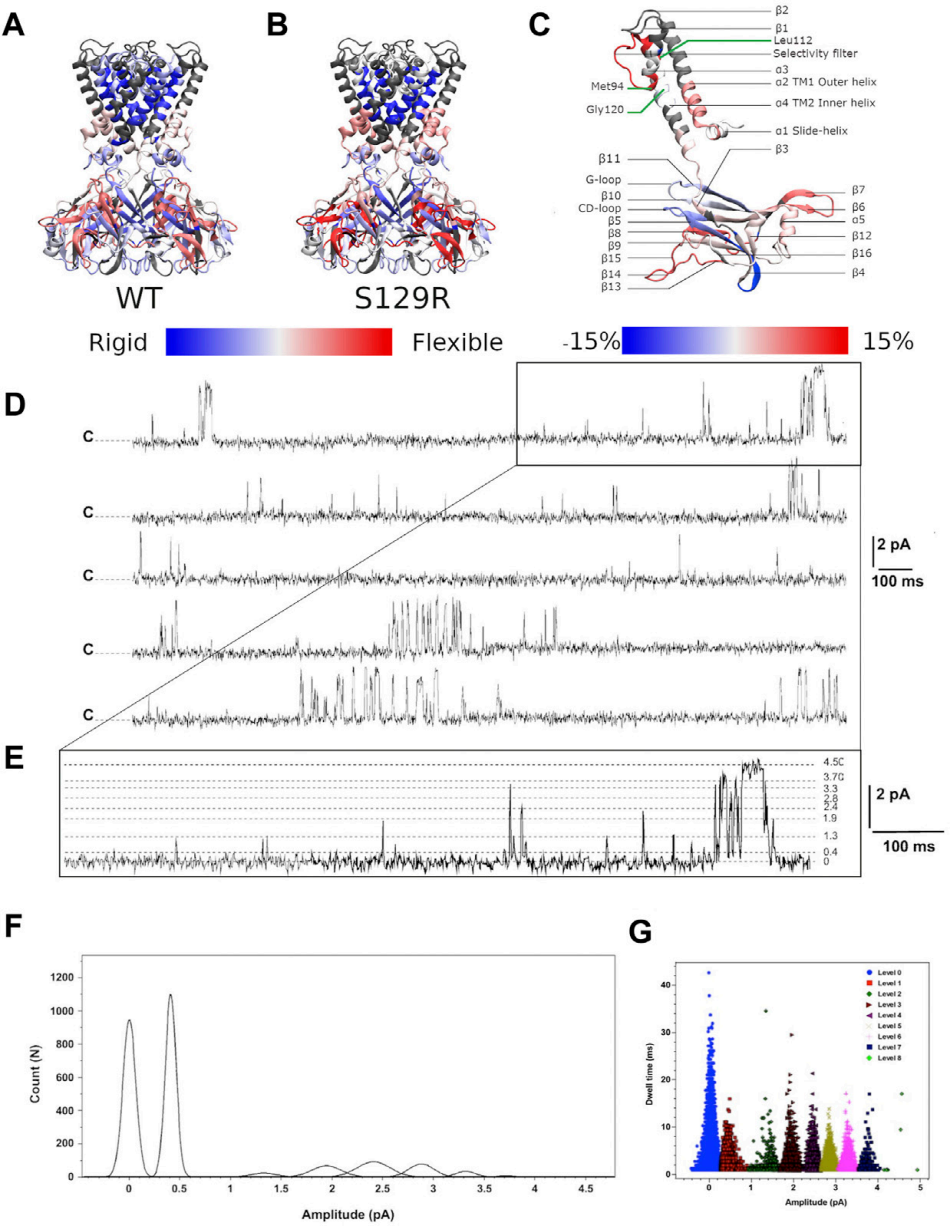
HDX-MS and single channel recordings HDX-MS rates of peptides reported for **(A)** KirBac3.1 WT (Forest et al., 2016) and **(B)** the mutant S129R. Identified peptides are drawn with blue (low exchange and low flexibility) to red (high exchange and high flexibility) color according to their percentage of deuterium exchange after 1,200 s (scale of exchange shown at the bottom of the figures). **(C)** Nomenclature of secondary structures and rate of exchanges between the mutant S129R and KirBac3.1 WT. Scale of deuterium exchange rate is shown at the bottom of the figures, same color code as for A and B. Red appears when S129R is more flexible than WT and blue when S129R is more rigid than WT **(D)** Single channel recordings from KirBac3.1 S129R channels, traces obtained from 6 min consecutive recordings at +150 mV holding potential. Closed state is labeled c **(E)**, an enhancement of the upper trace to show the multiple sub states induced by the S129R mutant **(F)** amplitude histogram fits (Gaussian) of all events for the selected levels gating between closed and open states during 6 min **(G)** S129R channels gate with multiple subconductance states. Dwell time for all events at 150 mV test potential is plotted for all current levels.

We investigated the protein conformational flexibility of the S129R mutant protein (open state) using HDX-MS. This technique is based on the exchange of deuterium atoms at the amide backbone of a protein, reflecting its conformational dynamics, followed by proteolytic digestion and spectrometry analysis. HDX has been widely used on soluble and membrane proteins (Forest et al., 2016). HDX was performed on the purified KirBac3.1 S129R mutant protein in the presence of detergent (Figure 2B). We have established in previous work that the presence of detergent does not affect conformational changes of the KirBac channel (Gupta et al., 2010; Fagnen et al., 2020). The results were compared to those of WT for which the same detergent was used [(Fagnen et al., 2020) and Figure 2A], the comparison is shown in Figure 2C where red shows a S129R segment more flexible compared with the same segment in WT. The Optimized conditions resulted in sequence coverage of 86% with nepenthesin (Fagnen et al., 2020). However, this enzyme did not allow covering the regions 57–87 (top half of the TM1 and the beginning of the pore helix), 143–147 (*β*3) and 195–203 (second half of the *β*7), for nomenclature see Figure 2C. Deuterium incorporation was monitored as a function of time for each peptide generated from the S129R mutant (Supplementary Figure S1).

#### Comparison Between the KirBac3.1 WT (Closed State) and S129R Mutant (Open State)

Our data shows that the most flexible regions for KirBac3.1 S129R are the loops extending outside the CTD (aa 271–285 between *β*14 and *β*15, in red Figure 2B; see also Figure 2C for the nomenclature). This external loop is subjected to the swinging movement during the gating (Fagnen et al., 2020). If we compare the closed state of KirBac3.1 and the open state of KirBac3.1 S129R, the latter shows slightly more pronounced flexibility with a maximum value for the HDX of 68% ± 2.2 (Mean ± S.D *n* = 3) against 59.1% ± 2.5 for the closed state as shown in Figure 2C.

#### Structural Flexibility of the Transmembrane Domain During the Gating

The largest change in the deuteration exchange percentage in the S129R mutant (35 vs. 21%, compared with WT) is observed for the Thr93-Leu112 peptides [end of *α*3 (pore helix), selectivity filter and top of *α*4 (TM2)], a feature described previously (Gupta et al., 2010). This includes the Met94 which is located towards the base of the pore-loop helix and packs closely with the Gly120 in TM2. Based on deuterium exchange percentages, the inner helix in the mutant S129R appears more flexible than in WT. At the TM1 level, there is an increase in deuteration, particularly at the bottom of the external helix, of 53.7% ± 2.2 against 45.2% ± 1.3 (Figure 2C).

#### Cytoplasmic Domain

These domains do not remain static during gating, and conformational changes should occur as the channel opens and closes. For both closed and open states, the greatest flexibility is found at the external loop. The KirBac3.1 cytoplasmic domain consists of two major *β*-sheets, one (which we refer to as *β*I, includes the large *β*6, *β*10, and *β*11), that is tilted about 45° to the membrane plane, and a second referred to as *β*II (which includes the shorter *β*3, *β*5, and *β*9) is approximately parallel to the pore axis as described in (Wang et al., 2012). Our flexibility measure shows that the main *β*I sheet is more rigid than the other major *β*II sheet for both the WT (closed) and S129R mutant (open). The cytoplasmic domain’s interior is more rigid than the exterior, with the highest values of flexibility for the open state.

On the contrary, the G-loop, located next to the CD loop is slightly less flexible in the S129R mutant (open state) (9% ± 1.2 against 12% ± 0.8 in the close state). The G-loop has been described as very flexible (Bichet et al., 2003; Pegan et al., 2005; Nishida et al., 2007; De Zorzi et al., 2013). The five amino acids, 162 to 174 of the CD-loop, are also more rigid in the S129R mutant (open state) than in the WT (closed state) (49% ± 2.3 vs. 58% ± 2.5). The decrease in deuteration in these two loops shows that they are involved in a network of interactions with neighboring amino acids, which are therefore less flexible. This was also found in (Gupta et al., 2010).

#### To Further Assess the Structural Deviations of These Two States

we compared the root mean square displacements (rmsd) of the residues (equation given in *Materials and Methods*) calculated over the MDeNM relaxed structures of KirBac3.1 WT (closed) and KirBacS129R (open). The results are shown in Supplementary Figure S2. Minor rmsd values were observed in the closed state, particularly in the transmembrane region. For both structures, the smallest rmsd values (less than 2.8 Å) are in the transmembrane region. The open state shows slightly higher values, particularly in the region of TM1, the pore helix, and the bottom of TM2 with a high value at the position of the S129R mutation. The cytoplasmic domain exhibits higher rmsd values particularly the external loop, which reaches 5.8 and 5.2 Å, for the open and closed states, respectively. This is in agreement with HDX-MS data, for which the highest flexibility is in this region.

### Current Recordings of KirBac3.1 in Planar Lipid Bilayers

When reconstituted into black lipid membranes, the KirBac3.1 S129R channels exhibit significant gating activity as shown by current recordings for 6 min at +150 mV (Figure 2D). As reported for the KirBac3.1 WT and KirBac 1.1, the KirBac 3.1 S129R gates with multiple subconductance states (Cheng et al., 2009; Clarke et al., 2010). The amplitude values for these levels are 0.40 ± 0.001, 1.32 ± 0.002, 1.94 ± 0.003, 2.41 ± 0.001, 2.88 ± 0.002, 3.31 ± 0.001, 3.71 ± 0.002, and 4.50 ± 0.007 pA (Figure 2E). Fits of Gaussian distributions of amplitude histograms resulted in a single channel current level of 4.5 ± 0.007 pA, corresponding to a conductance value of 30 pS (Figure 2F). A value similar to that has been obtained for KirBac 3.1 WT (47 pS) (Fagnen et al., 2020). KirBac S129R gates with subconductance level activity that increases the Po to levels as high as 44.05 ± 2.6% (Mean ± S.D. *n* = 16,107, number of events). Plotting dwell time vs. current amplitude of all the events shows that the S129R mutant gates with more subconductance levels, than the WT, that are contributing to the overall Po as follow: 7, 3, 11, 9, 5, 6, 4, and 1%, respectively, (Figure 2G). We have already shown that WT channels gate with only two subconductance levels (1 and 2 pA current amplitude) (Fagnen et al., 2020).

### Theoretical Results

The theoretical results presented in this section are based on MDeNM (Molecular Dynamics with excited Normal Modes) simulations in which different linear combinations of a selected set of normal modes (NMs) related to the opening/closing of the channel are excited in molecular dynamics (MD) simulations (Costa et al., 2015). Through such a combined use of both methods, MDeNM allows a realistic exploration of the normal mode space relevant for the opening/closing mechanism taking into account the full environment (membrane, water, ions) of the protein at the ambient temperature. Standard MD simulations were thereafter carried out on a uniformly distributed set of structures obtained from MDeNM, which provided a reasonably good estimate of the populations of open/closed (and partially open) states. MDeNM is based on covering uniformly without any bias the whole normal mode space defined by a selected set of low frequency modes. In our study the normal modes that were chosen are all those that are involved in the opening/closing motion of the channel. Therefore, the open and closed states were equally and uniformly sampled. The tests that were done in the original article (Costa et al., 2015) has shown an extensive not biased sampling, giving a good estimation of the probabilities of different states.

### Constriction Points Along the Channel in the KirBac3.1 WT and the S129R Mutant

**FIGURE 3:**
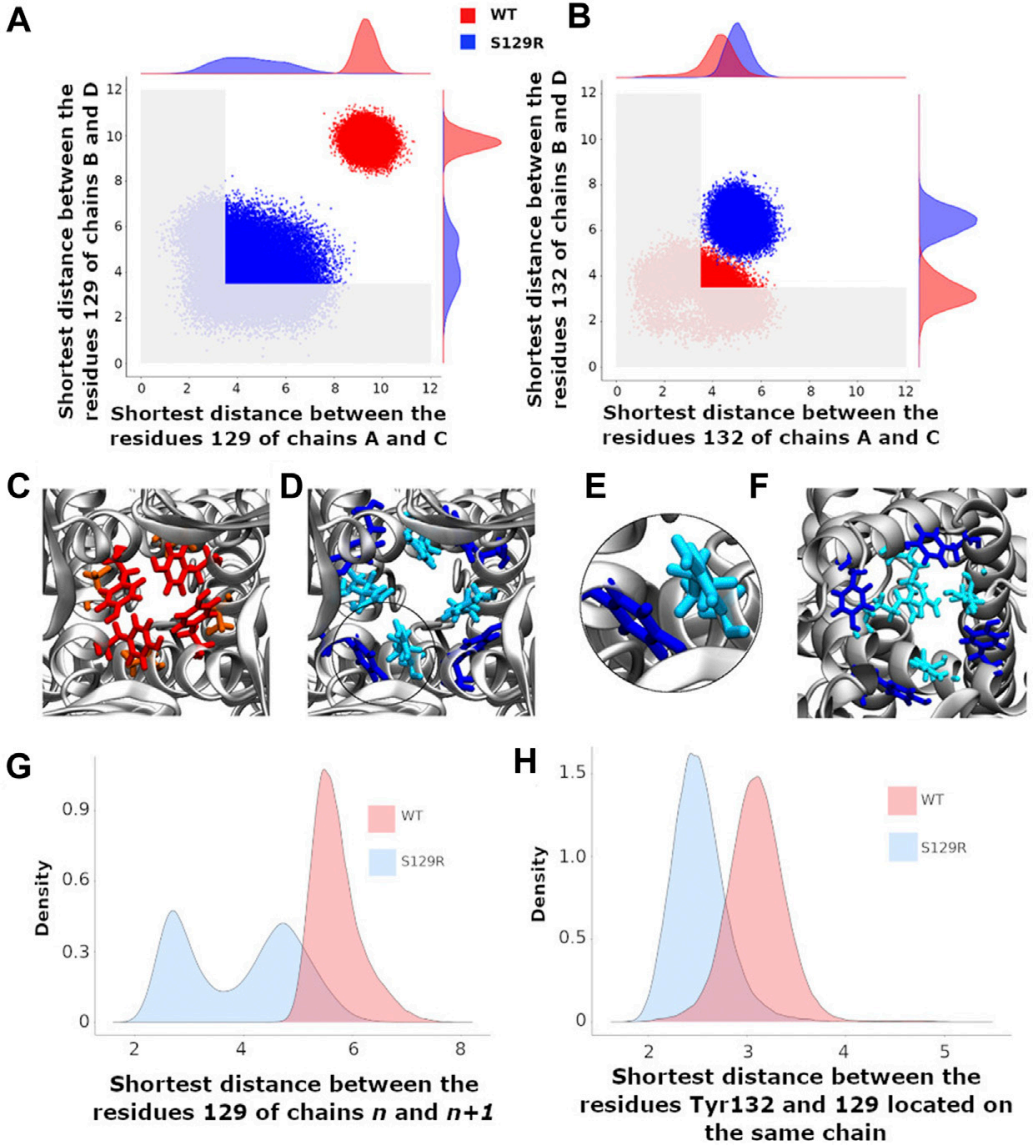
Gating at the mutation S129R and at the constriction point Tyr132 **(A)** Scatter plots of the shortest distances between the chains B and D and between A and C at the level of the residue 129. The shortest distance between two residues is that between their respective atoms including the hydrogens; **(B)** Scatter plots of the shortest distances between the chains B and D and between A and C at the level of the residue Tyr132. A,B) Red and blue points correspond to the KirBac3.1 WT and KirBac3.1 S129R, respectively. The gray area delimits the region where the channel is closed **(C–F)**: representative relaxed structures; **(C)** Locations of the residues Tyr132 (red) and Ser129 (orange) in KirBac3.1 WT; **(D)** Locations of the residues Tyr132 (dark blue) and Arg129 (cyan) in KirBac3.1 S129R; **(E)** Zoom on the Van der Waals contacts between Tyr132 and Arg129 in KirBac3.1 S129R; **(F)** Locations of the residues Tyr132 (dark blue) and Arg129 (cyan) pointing toward the center of the channel in KirBac3.1 S129R; **(G)** Histograms of the shortest distance between residue 129 of the chain *n* and the residue 129 of the chain *n*+1; **(H)** Histograms of the values of the shortest distances between the residues 129 and Tyr132 from the same chain. The histograms in panels **(G,H)** were established by taking into account all the chains.

In this work, a “closed” state is defined by a conduction pathway which is sterically occluded and an “open” state in which the pathway is sufficiently wide to accommodate at least a non hydrated potassium ion. The channel encompasses the region between residues 121 and 133. The constriction points in this region for the WT are located at the levels of Leu124 and Tyr132 (Figure 1B). To have a dynamical view of the relaxed structures obtained in the MD simulations (that follow the MDeNM), we calculated the shortest atom-atom distance (including hydrogens) between the same residues that are at the opposite chains at a given constriction level (Tyr132 or S/R129). The distances were calculated on KirBac3.1WT (closed state) and the KirBac3.1 S129R (open state) (Figure 3A,B, respectively). The average shortest distance at the level of Ser129 in KirBac3.1 WT is 9.53 Å (SD = 0.47 Å) (Figure 3A in red) and that at the level of Arg129 in S129R mutant is narrower with a value of 4.46 Å (SD = 1.28 Å) (Figure 3A in blue). This is a marked decrease in the channel diameter at the level of the mutation. Interestingly, the mutation at residue 129 introduces another constriction point in the channel which should not allow the K^+^ ion to pass easily. Figure 3 shows a set of distances situated in the gray zone which is indicative of a closed state.

**FIGURE 4:**
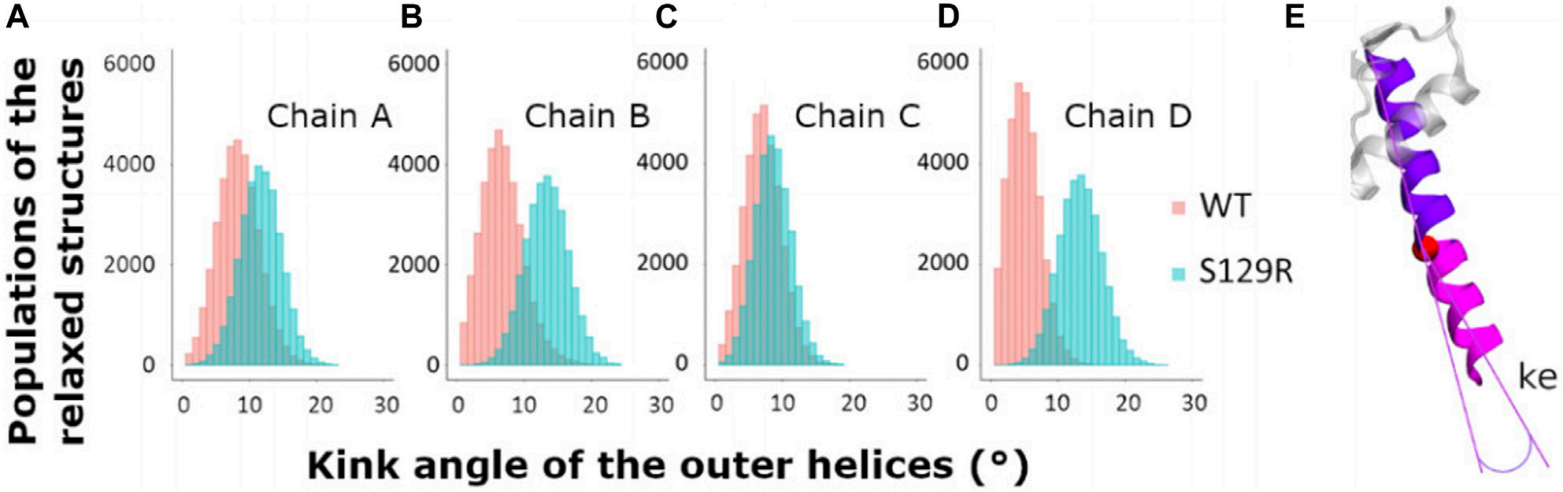
Kink of the outer helices, TM1. Histograms of the values of the kink of the external helices during the relaxed simulations of molecular dynamics after MDeNM. The structures populations of KirBac3.1 WT are represented in red and the structures of KirBac3.1 S129R in blue **(A)** Kink of the outer helix of the chain A **(B)** Kink of the outer helix of the chain B **(C)** Kink of the outer helix of the chain C **(D)** Kink of the outer helix of the chain D **(E)** Representation of the outer kink angle (ke).

Tyr132 has been described as a constriction point in KirBac3.1 WT confirmed with an average shortest distance obtained of 3.72 Å (SD = 0.87 Å) (Fagnen et al., 2020). In the KirBac3.1 S129R mutant, the average shortest distance, at this constriction point is larger with a value of 5.75 Å (SD 0.87 Å) (Figure 3B points in blue and Figure 1B in orange). This point of constriction is therefore released. The Leu124 constriction point is also in an open configuration in the KirBac3.1 S129R mutant (Figure 1B in brown). We provided in Supplementary Table S1 the radius values of the pore (computed with the HOLE program) at the constriction levels, as well as the kink angle values of the TM1 for the KirBac3.1 WT and KirBac3.1 S129R crystallographic structures, and KirBac3.1 S129R simulated structures in one of their closed or open conformations. It is worth to notice that the pore at the level of the mutation can adopt a narrower radius in a representative MDeNM structure comparatively to its X-ray structure. We also notice that at the level of the residue 132 we have a larger pore radius for the simulated closed structure than in the X-ray structure. The kink angles of TM1 in three chains out of four are also larger in the chosen simulated open structure than in the closed one, but their mean values (Figure 4) are all larger in the mutant than in the WT, a trend similar to what is observed when comparing both X-ray structures.

### Interactions of the Residue 129

We investigated the interactions of residue 129 with its neighboring residues and the changes in the interaction network caused by the mutation in all the relaxed MDeNM structures (Fagnen et al., 2020).

The probability density of the distances between two adjacent Ser129 in WT showed a single peak around 5.9 Å, the two residues being quite distant (Figure 3G, in red). Note that higher density of probability means more favorable interactions. For the mutant, the distances between two adjacent Arg129 are distributed into two populations centered around 2.7 and 4.8 Å (Figure 3G, in blue). The first peak corresponds to a close interaction between the two arginine heads which point towards the center of the channel and therefore obstructing it (Figure 3F in cyan), the second representing more distant residues similar to KirBac3.1 WT. The interaction energy computed between pairs of Arg shows that those belonging to opposite chains can interact favorably as shown in the scatter plot of the interaction energy *vs.* the shortest distance in Figure S3 computed for the ensemble of the MDeNM relaxed structures. It is seen that interaction energies can reach values close to −4 kcal/mol. The representative structure corresponding to the lowest interaction energy is also displayed in this figure. Although Arg has a positive charge it was shown that they can interact favorably between them adopting different orientations (Pednekar et al., 2009).

Note that the distribution of shortest distances depend on which chains are considered. Indeed, the crystallographic structures of KirBac can exhibit four-fold symmetry, but more often two-fold symmetry or even no symmetry (Clarke et al., 2010; Bavro et al., 2012; De Zorzi et al., 2013). Moreover, the side chains in these crystallographic structures are slightly asymmetric.

We investigated the interaction between the mutated residue and Tyr132 which constitutes a region of constriction along the channel in WT (Figure 3H). The most populated shortest distance is greater for WT (3.3 Å) than for mutant (2.5 Å). The contact between the two residues for the mutant is shown in Figures 3D,E.

In the closed state, residue 132 points towards the center of the channel and obstructs the passage of the K^+^ ion (Figure 3C in red). In the mutant, Tyr132 is displaced from the channel’s center coming in contact with Arg129, as shown in Figure 3D (See also Supplementary Figure S4 for details).

The interaction energies between the Arg129 and Tyr132 as a scatter plot are given in Supplementary Figures S5, S6 with the molecular graphics of the most favorable structures. They show that they can interact strongly with an interaction energy around −10 kcal/mol in the case of Arg129B and Tyr132C forming a hydrogen bond between them (Supplementary Figure S6), and around −5 kcal/mol between Arg129B and Tyr132B. Interestingly, the Arg129 residues in all four chains point either to the center of the channel and thus block the passage of K^+^ ion (Figure 3F) or interact with the aromatic ring of Tyr132 residues (Figure 3D).

### Open and Closed State Populations

Four channel-gating states can be defined based on the open or closed conformation of the two main constriction points (Leu124 and Tyr132) as observed in relaxed structures [for more details, see (Fagnen et al., 2020) Supplementary Figure S7]: 1) Fully open state, sufficiently wide to accommodate at least a non-hydrated K^+^ ion, that is, when the shortest diametrically opposed inter-chain atomic distances at the two constriction points are greater than the ionic diameter of K^+^ [diameter of K^+^ ion considered 3.5275 Å (Huang and MacKerell, 2013)]; 2) fully closed state, when both distances are less than the ionic diameter of the potassium ion; 3) partially open state 1, when the gate at residue Leu124 is open, and the gate at residue Tyr132 is closed; 4) partially open state 2 when the gate at residue Tyr132 is open, and the gate at residue Leu124 is closed. Considering the previous observation showing that the side chain of the mutant Arg129 can point towards the center of the channel and constitutes another constriction point, we therefore added another partially closed configuration at the level of Arg129.

We analyzed 34,086 relaxed structures issued from MDeNM simulations for KirBac3.1 WT and 29,600 for KirBac3.1 S129R to have important information on these states’ populations. The populations of the different WT and mutant states are shown in Table 1, indicating that the fully open state in KirBac3.1 WT is only populated by about 6.8%. Such a low value is consistent with the population obtained by previous electrophysiological experiments (Fagnen et al., 2020). In contrast, the S129R mutant is mostly open by about 53%, the Arg129 keeping the two Leu124 and Tyr132 restriction points, always open. Interestingly, the Arg can adopt two conformations: one in which it interacts directly with Tyr132, and the other where it points to the channel’s center obstructing it. Therefore, the closed state of the mutant depends only on the conformation of Arg. Ironically, the mutated residue, which was introduced in the protein to force the channel to open, by trapping the bundle crossing in an open conformation, causes some obstruction to the potassium ion's passage.

**TABLE 1:** Populations (in percentage) of different opening states in the relaxed structures of KirBac S129R obtained through MDeNM simulations and single channel recording; comparison with KirBac3.1 WT is shown.

Opening types	KirBac3.1 WT (%)	KirBac3.1 S129R (%)
Fully open	6.8	52.8
Fully closed	50.2	0.0
Gating 124 open, gating 132 closed	28.8	0.0
Gating 132 open, gating 124 closed	14.2	0.0
Gating 129 closed	0.0	47.2
Current recordings of KirBac3.1 in planar lipid bilayers	9.9 (±1.3, *n* = 1803) (ref 14)	44.05 (± 2.6% mean ± S.D. *n*= 16,107)

### Structural Modifications Between the Closed (KirBac3.1 WT) and Open (KirBac3.1 S129R) States

#### Kink of the Outer Helix TM1

The TM1 outer helices’ kink angles were calculated to determine the extent to which their bending is involved in the channel’s opening. We compared the kink angles for the TM1s for KirBac3.1 WT and S129R, which are given in Figure 4.

We calculated the kink of the outer helix on all the relaxed structures. The mean values of the kink of TM1 for the chains A, B, C, and D of KirBac3.1 WT are respectively of 8.39° (SD = 3.09°), 6.51° (SD = 3.12°), 7.04° (SD = 2.70°), and 4.97° (SD = 2.39°) while the mean values for KirBac3.1 S129R are 11.70° (SD = 3.03°), 13.33° (SD = 3.18°), 8.59° (SD = 2.66°), and 13.308° (SD = 3.18°). These results highlight that the presence of the mutation S129R on TM2 has a knock-on effect on TM1, triggering a greater kink of this outer helix as noticed in the cryo-EM analysis (Paynter et al., 2010).

#### Rotation of the Cytoplasmic Domain

**FIGURE 5:**
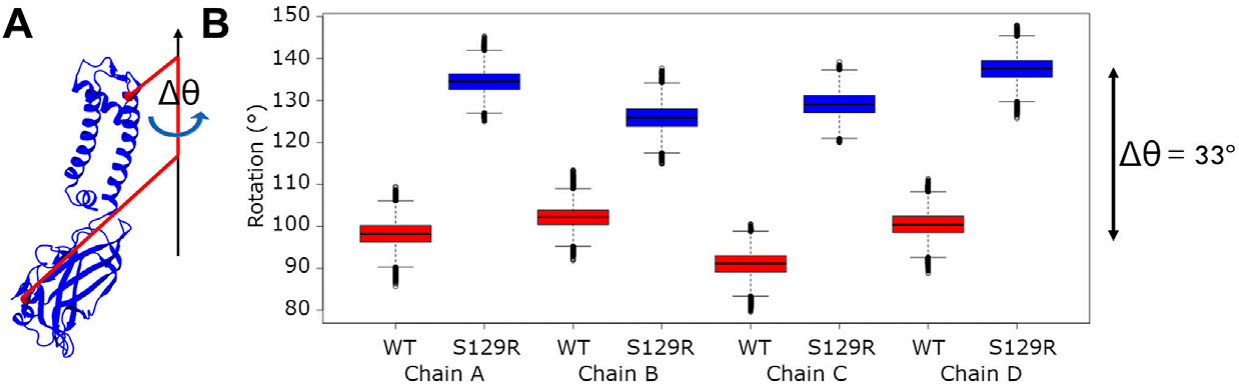
Rotation of the cytoplasmic domain **(A)** Definition of the cytoplasmic domain rotation angle of a given chain; **(B)** Boxplots of the rotation angle’s values of the cytoplasmic domain for each chain for KirBac3.1 WT and KirBac3.1 S129R. The angles were computed on all the relaxed structures of KirBac3.1 WT (red) and KirBac3.1 S129R (blue). The middle line of each box corresponds on the median values. The variation of the 33° between the WT and mutant were obtained from these average values.

Motions of the cytoplasmic domain that couple ligand binding to the gating of the channel have been thoroughly investigated and various models have been proposed. From the KirBac3.1 S129R mutant’s crystallographic data, a model described as “twist to open” has been proposed, on which a rotation of about 25° of the CTD around the central axis of the channel perpendicular to the membrane is crucial to allow gating. This is why we performed a thorough examination of the angle of rotation of each chain’s cytoplasmic domain around the central axis on all the relaxed structures (See the definition of the rotation angle Figure 5A).

We compared the rotation angles of the cytoplasmic domain of the KirBac3.1 WT and KirBac3.1 S129R, they are given in Figure 5B. The mean cytoplasmic domain rotation values are 97.95° (SD = 5.13°) and 131.78° (SD = 5.41°) for WT and mutant, respectively. An average difference of 33° is observed between the two systems. This is to be compared with the data obtained from crystallographic structures, which show a difference of the cytoplasmic domain rotation angle of 30° between the KirBac3.1 WT (closed state) and KirBac3.1 S129R (open state) (Bavro et al., 2012). It can be observed that both experimental and *in silico* studies are very comparable. The structures obtained by MDeNM appear very stable with very small variations.

**FIGURE 6:**
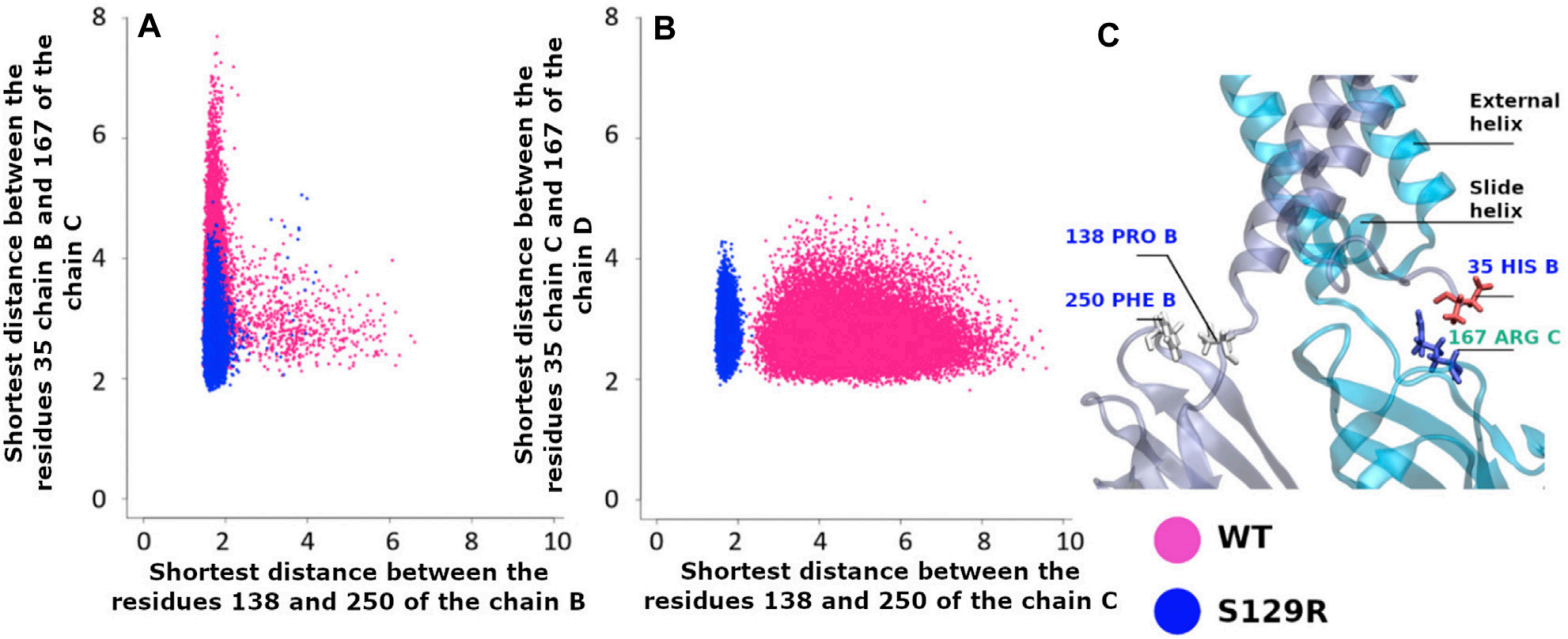
Interaction between the cytoplasmic and the membrane interface. Pink points are for KirBac3.1 WT, blue points for the S129R mutant **(A)** Shortest distance between the slide-helix (residue Asp35 Chain B and the CD-loop (residue Arg167 Chain C) vs. the shortest distance between the linker (residue Pro138 Chain B) and the G-Loop (residue Phe250 Chain B); **(B)** Shortest distance between the slide-helix (residue Asp35 Chain C) and the CD-loop (residue Arg167 Chain D) vs. the distances between the linker (residue Pro138 Chain C) and the G-Loop (residue Phe250 Chain C); **(C)** Location of the interactions.

To study the S129R mutation effect on the interaction between the cytoplasmic domain and the membrane interface, we calculated two distances: 1) between His35 on the slide helices and Arg167 (CD-loop on the CTD); 2) between Pro138 (the linker between the transmembrane and the cytoplasmic domain) and Phe250 (G-loop on the CTD). The G-loop has been described as being mobile during gating in molecular dynamics studies (Bernsteiner et al., 2019; Li et al., 2019) and X-ray data (Pegan et al., 2005). The location of residues is indicated in Figure 6C.

The structures resulting from the dynamics highlight the differences between WT and the mutant. Figure 6A shows that the interaction between Pro138 and Phe250 (chain B) is stronger in S129R compared to WT. The distances between the amino acids are from 1.91 to 5.06 Å in the S129R mutant (in blue) and from 1.82 to 7.70 Å in KirBac3.1 WT (in red) predominantly closed (93.2% of the structures). The same trend is observed for the chain C, shown in Figure 6C, as they do not exceed 2.34 Å while the range of values for WT extends over 9.58 Å (Figure 6B). The distances between the residues His35 Chain B and Phe167 Chain C are also lower in S129R

The S129R mutant shows a stronger interaction between the cytoplasmic domain (G-loop and C loop) and the membrane interface (slide helix and linker) and a greater stability.

## Conclusion

Our MDeNM simulations on S129R show clearly that the mutation leads to a greater opening probability of about 52.8% compared to 6.8% in the WT, which is corroborated by functional data from single channel recordings 44.05 (±2.6% Mean ± S.D. *n* = 16,107) compared with 9.9 (±1.3, *n* = 1803) in the WT. In this study, we observed the opening of the constriction points in the channel and the inherent motions of KirBac3.1 S129R associated with the gating in the absence of K^+^ inside the pore. Our *in silico* results, in the estimation of open state population, carried out in the absence of K^+^ ions, are very similar with experimental electrophysiological accounting of these ions. The gating is therefore mainly linked to the intrinsic dynamical properties of the channel and not dependent only on the presence of the K^+^ ions. The presence of the Arg mutation triggers the release of the two constriction points that existed in WT protein, but at the same time, this residue can block the passage of the K^+^ ion through the channel. Indeed, Arg can adopt two conformations, pointing either towards the channel’s center or standing parallels to the channel and interacting with Tyr132. This explains why the opening probability is only 52.8% and not 100%. Also, the transmembrane external helices (TM1) show a more pronounced kink and flexibility in the case of the S129R mutant in agreement with our HDX-MS experiments. In addition, the contacts between Pro138 and Phe250 (G-loop), and the inter-chain contact between His35 (slide-helix) and Arg167 (CD-loop) are stabilized, and the mutant shows a greater stability.

One of the conclusions of this study is that care must also be taken when selecting replacement residues since this could affect importantly the structural and dynamical behaviour of the system under consideration as it is the case here.

## Materials and Methods

This article focuses on understanding the structural behavior of KirBac3.1 S129R, making the comparison with the KirBac3.1 WT necessary to detect its specific aspects. The data and details concerning the latter were described in our previous article (Fagnen et al., 2020).

### Protein Expression and Purification

Same construct as for the protein used for the structure determination was used (Bavro et al., 2012). Protein expression and purification of this mutant channel were performed as outlined before (De Zorzi et al., 2013). Briefly, after cell disruption by French press, the protein was solubilized with 45 mM DM (Decyl *β*-D-maltopyranoside), centrifuged, and the supernatant was loaded onto a Co2+ affinity column. The protein was promptly purified on a Superdex 200 column pre-equilibrated with 2 mM TriDM buffer. Concentrated preparations (1–2.5 mg/ml) of purified proteins (>95% purity, judged by SDS-PAGE) were stored at −80°C in a buffer containing 20 mM Tris, pH 7.4, 150 mM KCl and 0.2 mM TriDM.

### Pepsin Digestion, Hydrogen/Deuterium Exchange Coupled to Mass Spectrometry and HPLC Peptide Separation and Mass Spectrometry of Peptides.

These experiments were performed as outlined before (Fagnen et al., 2020). Briefly, all protein digestions in solution were performed in an ice bath at 0°C. Protease solutions were prepared in 500 mM glycine (pH 2.2). KirBac3.1 S129R protein samples were digested in the same buffer for 2–5 min using a protease/substrate ratio of 1:1 or 1:10 (wt/wt) for pepsin and nepenthesin, respectively, either in solution or immobilized on a resin. The increase in digestion time did not affect the proteolysis. HDX-MS reactions were carried out on KirBac3.1 S129R at a protein concentration of about 10 μM. The reaction was initiated by a 10x dilution of the protein samples (10 μl) into a deuterated buffer containing 50 mM KCl and 0.2 mM TriDM. The time course of the HDX was followed over a 20-min period by sequential withdrawing 120 μl of deuterated samples, which were immediately added to 26 μl of quenching buffer (8 M guanidium chloride, 500 mM glycine HCl, pH 2.2), rapidly mixed, and flash-frozen in liquid nitrogen. After protease digestion in solution or on column in an ice bath at 0°C, the peptides were loaded onto a peptideMicroTrap (Michrom Bioresources) column and washed with 0.03% TFA in water (HPLC). They were then separated on a reversed-phase C12 column (1 × 50 mm, Jupiter; Phenomenex) using a linear gradient of 15–45% (vol/vol) of solution B (CH3CN 95% and TFA 0.03%) during 26 min. The tandem MS (mapping) analyzes were performed on an ion trap mass spectrometer (Esquire 3000+; Bruker Daltonics) to identify the peptides after their separation on HPLC. Accurate mass measurements and local deuteration kinetics analysis were performed on a time-of-flight (TOF) mass spectrometer (6210; Agilent Technologies) equipped with an electrospray source. Each deuteration experiment was performed in triplicate. Data were processed as described in (Fagnen et al., 2020).

### Electrophysiology

An Orbit mini was used (Nanion, Germany, horizontal planar lipid bilayer system), where two aqueous chambers (150 µl) are separated by a partition with a 150-µm hole where the lipid bilayer is formed by 1,2-diphytanoyl-sn-glycero-3-phosphocholine (DPhPC,15–50 pF). The lower chamber contained 150 mM KCl, 10 mM MOPS, pH 7.4. After membrane bilayer formation, the upper chamber solution was changed to 150 mM KCl, 10 mM MOPS pH 8.1 µl of purified KirBac3.1 S129R (90 μg/ml) in DDM (n-DoDecyl-β-D-maltoside) detergent (0.015%) was added to the upper chamber to a preformed bilayer. Currents were recorded using Elements Data Reader (Nanion, Germany) and analyzed using Clampfit (Axon Instrument Inc., United States) software, sampled at 100 µs and filtered at 1.25 kHz. Recordings were performed at 24°C.

### Molecular Modeling

KirBac3.1 was modeled in two different states, the closed one, modeled from the PDB structure of the wild type, 2WLJ (at 2.60 Å atomic resolution) described also previously (Fagnen et al., 2020), and the open one, modeled from the PDB structure 3ZRS (KirBac3.1 S129R at 3.05 Å resolution). KirBac3.1 S129R had missing C and N-terminal fragments, so the modeled structure describes the protein from residue 35 to 295; other missing atoms were rebuilt using the CHARMM program. We applied the P42_1_2 symmetry to the 3ZRS PDB structure using the Protein Interface Surface and Assemblies software (Krissinel and Henrick, 2007) to build the tetramer. We applied the “Orientation of Proteins in Membranes” (OPM) software (Lomize et al., 2012) and the protein was then included into a 114.566 × 114.566 × 128.572 Å^3^ water box containing 36,629 water molecules with 150 mM KCl, and an 1,2-dioleoyl-sn-glycero-3-phosphocholine (DOPC) membrane using CHARMM-GUI (Jo et al., 2008; Lee et al., 2016).

### Normal Modes

Structures were minimized using the steepest descent (SD) and conjugate-gradient (CG) methods followed by Adopted Basis Newton Raphson (ABNR) algorithm. Harmonic restraints were applied during SD minimization, which were gradually reduced. Then the system was subjected to 50,000 CG steps without restraints followed by ABNR minimization until a convergence of 10^–5^ kcal mol^−1^ Å^−1^ RMS energy gradient was reached. The first 200 lowest frequency normal modes, ranged in ascending order of frequencies, with all the atoms taken into account were computed using the iterative DIMB method (Mouawad and Perahia, 1993; Perahia and Mouawad, 1995) in CHARMM.

It was necessary to select the modes that contribute the most to the channel’s conformational changes to proceed with the MDeNM calculations. The channel was defined from residues 121 to 133. For each normal mode, the minimized energy structure was first globally displaced by 2 Å of RMSD. To select them, a process similar to the one used for the KirBac3.1 WT was applied (Fagnen et al., 2020). Firstly, the ten normal modes showing the highest variations at the level of the channel were retained. To evaluate these variations, the distances between the residues 125 of opposite chains were considered. Secondly, only the normal modes describing spherical and elliptical opening of the channel were taken into account. Redundant modes were excluded keeping only the lowest frequency normal modes. This protocol allowed us to select four modes that affect the most the gating of the S129R.

### Molecular Dynamics Using Excited Normal Modes

The Molecular Dynamics using excited Normal Modes (MDeNM) method (Costa et al., 2015), promotes large conformational changes while taking into account the coupling with local fluctuations. This method allows a larger exploration of the conformational space and the generation of a wide variety of different structures at a lower computation time, which would not have been possible using only the standard MD. The method consists first to achieve different linear combinations of the selected normal mode vectors such that the combined vectors describe different movements of a given region (here the channel region). They are chosen such that the displaced structures along them up to a given distance (1 Å) display a uniform distribution of local RMSDs between them (see ref (Costa et al., 2015) for more details).

In a second stage these directions are used in MD simulations for defining additional velocities oriented along these very directions, and corresponding to a given kinetic energy, that are added to the current MD velocities. Such a kinetic excitation was periodically repeated at a given period of time (called the relaxation time) for propagating the movement to larger distances and allowing the coupling with local motions. A sufficient number of excitations were applied to reach an energetically acceptable large displacement. The simulations were carried out independently for every NM combined vector, each of these simulations being called a replica simulation. For each replica 10 successive excitations were applied, each one corresponding to a 4 K rise of the overall temperature; the relaxation time between two excitations was 1 ps The numbers of replica used are 62 and 66 for KirBac3.1 WT and KirBac3.1 S129R, respectively.

#### Relaxation of the MDeNM Structures

Free MD simulations were carried out on MDeNM structures to relax them further energetically and release the excess kinetic energy that would have been accumulated during the excitations. These relaxation simulations were carried out on a limited number of representative structures obtained by clustering the MDeNM structures to save simulation time. The VMD clustering tool (Humphrey et al., 1996) was used to find at least 100 different clusters separated by a distance greater than an RMS threshold of 0.9 Å on the channel (from Met121 to Ala133). A representative structure was chosen for each cluster, which was the closest to the cluster’s average structure. Unique structures not belonging to any cluster were also selected. Overall, 114 and 99 clusters were obtained for KirBac3.1 WT and KirBac3.1 S129R, respectively. Each representative structure was subjected to a free MD simulation of 0.4 ns to release the excess kinetic energy and allow local movements to occur, amounting to a total of 39.6 ns for all the KirBac3.1 S129R’s structures considered. The simulations were carried out with NAMD v2.10 (Phillips et al., 2005) at constant temperature of 300 K and constant pressure of 1 atm using Langevin piston. Periodic Boundary Conditions and the Particle Mesh Ewald method were used for the electrostatic interactions. The motion propagation is driven by the Leapfrog Verlet algorithm. Concerning the non-bonded interactions, the cut-on and the cut-off were 10 and 12 Å, respectively. Charmm36 force field was used for the simulations. The parameters used were the same for the MDeNM simulations.

#### Analysis of Molecular Dynamics Simulations.

The results presented in this article are based on the relaxed structures from free MD simulations in which only the last three-quarters of the trajectories were kept, that represents 29,600 structures.

The shortest distance between two residues was calculated considering the distances between all their respective atoms including the hydrogens with the CHARMM software. The shortest distances calculated at the different levels of the channel and different pairs of residues were used to define the various open/closed states. Six shortest distances were considered: 1) between Leu124 of chain A and Leu124 of chain C, 2) between Leu 124 of chain B and Leu124 of chain D, 3) between Tyr132 of chain A and Tyr132 of chain C, 4) between Tyr132 of chain B and Tyr132 of chain D, 5) between Arg129 of chain A and Arg of chain C, 4) between Arg129 of chain B and Arg129 of chain D.

The kink of each of the outer helices is defined by the angle between the axis of the first part of the helix going from Trp46 to Leu56 and that of the second part going from Leu56 to Asp80.

The cytoplasmic domain rotation for each of the chains was calculated as the pseudo bonds’ dihedral angle defined by four successive points defined by Leu108, its projection on the central *Z*-axis passing through the channel, the projection of Ile266 on the same axis, and Ile 266 itself.

The kink and the dihedral angles were calculated using the CHARMM software.

The Root-Mean-Square Deviation of a given atom *i* (RMSD_i_) was computed over the ensemble of all the MDeNM relaxed structures for KirBac3.1 WT (34,086 structures) and KirBac3.1 S129R (29,600 structures), respectively. The RMSD_i_ is defined by the equation

$$RMSD_{i}=\sqrt{\frac{1}{N}\;\overset{N}{\underset{n=1}{\sum }}{\left|r_{i}\left(n\right)-\;r_{i}^{ref}\right|}^{2}}\;$$

where *i* is the atom number, *N* the total number of structures considered, $\;r_{i}\left(n\right)\;$ the position of the atom *i* in the structure *n*, $r_{i}^{ref}$ the position of the atom *i* in the reference structure. The RMSD of a given residue was calculated by averaging the RMSD_i_ of atoms belonging to the given residue.

## Data Availability

The original contributions presented in the study are included in the article/Supplementary Material, further inquiries can be directed to the corresponding authors.
