# Supplementary material for: Unexpected Gating Behaviour of an Engineered Potassium Channel Kir
Source: Front Mol Biosci. 2021 Jun 10;8:691901. doi: 10.3389/fmolb.2021.691901 (PMC8222812; doi:10.3389/fmolb.2021.691901)
Supplement: Supplementary file 1 [file DataSheet1.PDF]

Supplementary

Unexpected gating behaviour of an engineered potassium channel Kir

Charline Fagnen[1,2], Ludovic Bannwarth[1], Dania Zuniga[1], Iman Oubella[1], Rita de Zorzi[3], Eric Forest[4], Rosa Scala[5], Samuel Guilbault[5], Saïd Bendahhou[5], David Perahia[2] and Catherine Vénien-Bryan[1]

[1]Sorbonne Université, UMR 7590, CNRS, Muséum National d'Histoire Naturelle, Institut de Minéralogie, Physique des Matériaux et Cosmochimie, IMPMC, 75005 Paris, France.
[2]Laboratoire de Biologie et de Pharmacologie Appliquée, Ecole Normale Supérieure Paris-Saclay, Centre National de la Recherche Scientifique, Gif-sur-Yvette, France.
[3]Department of Chemical and Pharmaceutical Sciences, University of Trieste, Via Licio Giorgeri 1, 34127, Trieste, Italy
[4]University Grenoble Alpes, IBS, F-38044 Grenoble, France, CNRS, IBS, F-38044 Grenoble, France, CEA,IBS, F-38044 Grenoble, France.
[5]University Côte d'Azur, CNRS UMR7370, LP2M, Labex ICST, Faculté de Médecine, 06107 Nice, France.

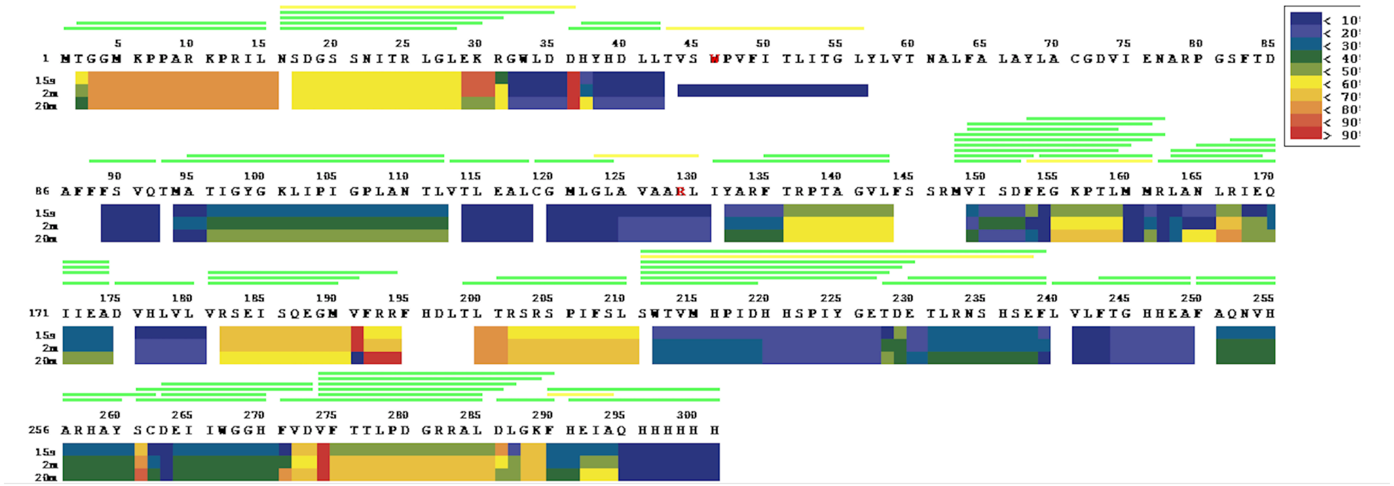

Figure S1: - Heat Map of the KirBac3.1 S129R. Deuteration maps of KirBac3.1 S129R peptides at different times up to 20 minutes deuteration. The color key indicates the HDX level.

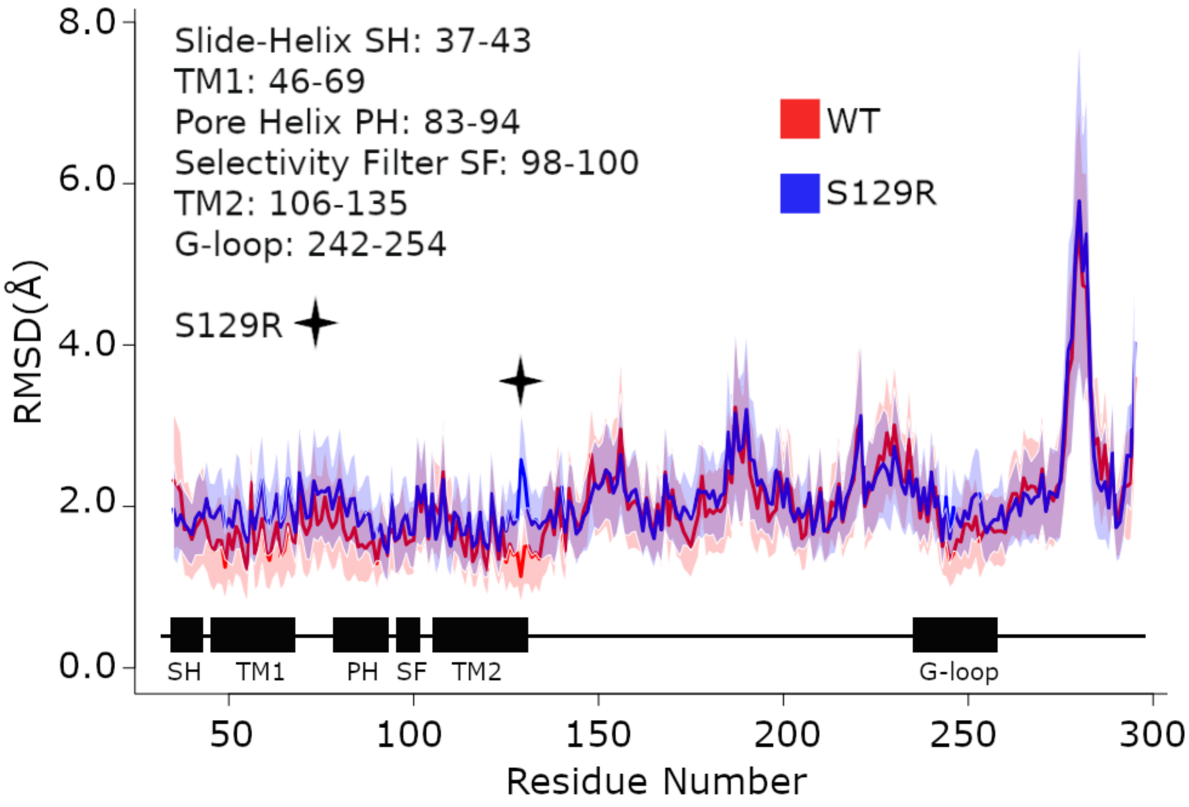

Figure S2: Root Mean Square Deviation per residue (see the equation in the main text) from the respective X-ray structures computed on the MDeNM relaxed structures for KirBac3.1WT and KirBacS129R, colored in red and blue, respectively. The standard deviations of RMSDs for both structures are indicated by the shaded zone with respective colors.

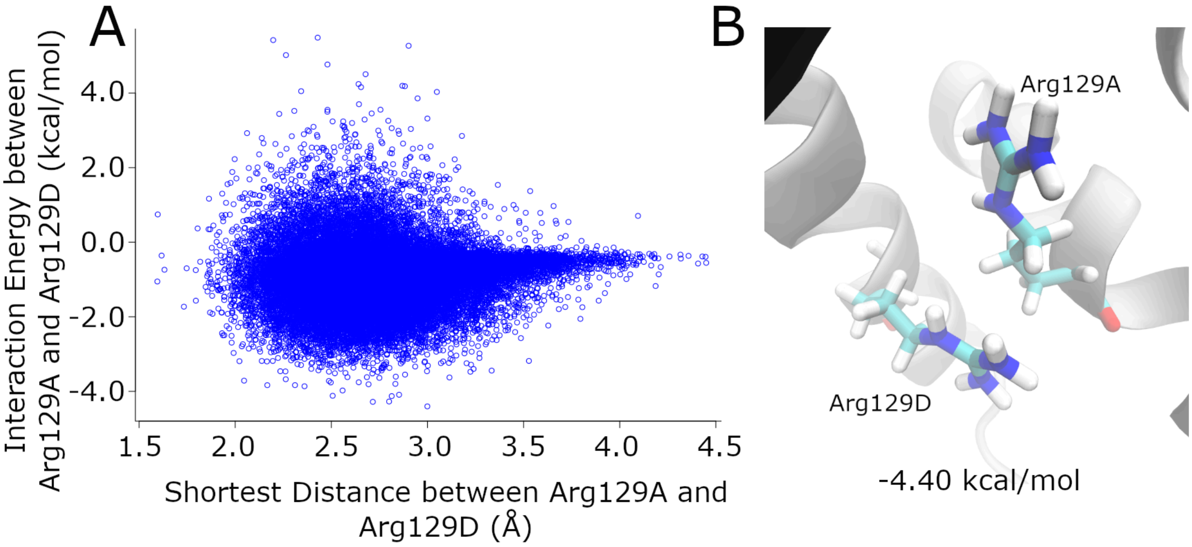

Figure S3: Interaction Arg129A-Arg129D A) Scatter plot of the interaction energy between Arg129A and Arg129D versus the shortest distance between these two residues. B) Atomic representation of the residues Arg129A and Arg129D at their more favorable interaction energy (-4.4kcal/mol).

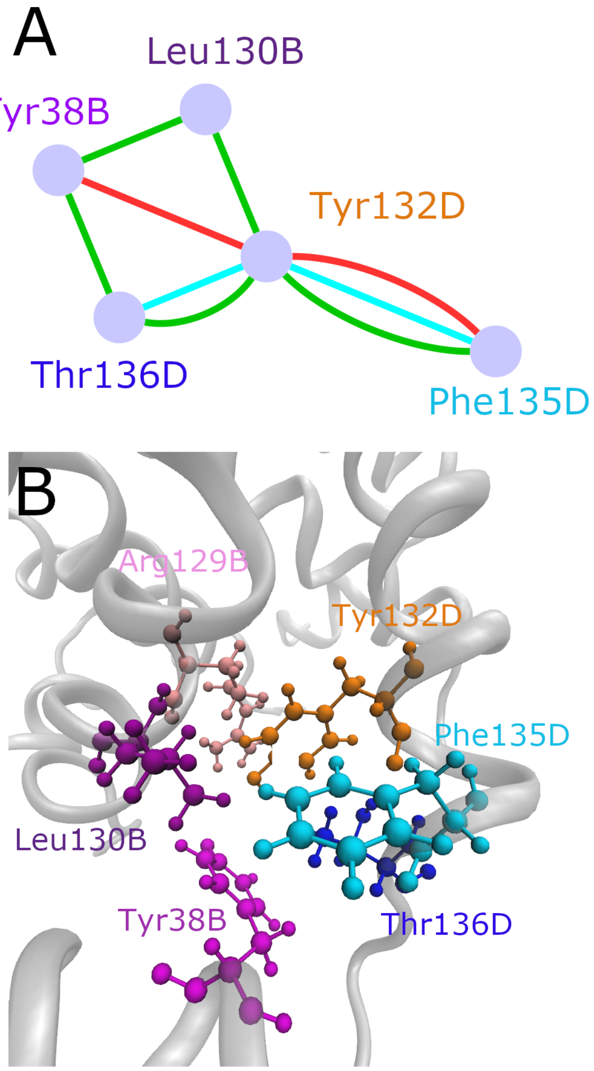

Figure S4: Interactions of the Tyr132 in KirBac3.1 S129R. A) Representation of the residue network of Tyr132 with the protein's residues. This network was generated using the RING server and displayed with Cytoscape software. The edges represented the Van der Waals interactions (green), hydrogen bonds (blue), and π-stacking (red). B) Location of the residues involved in the residues network of Tyr132.

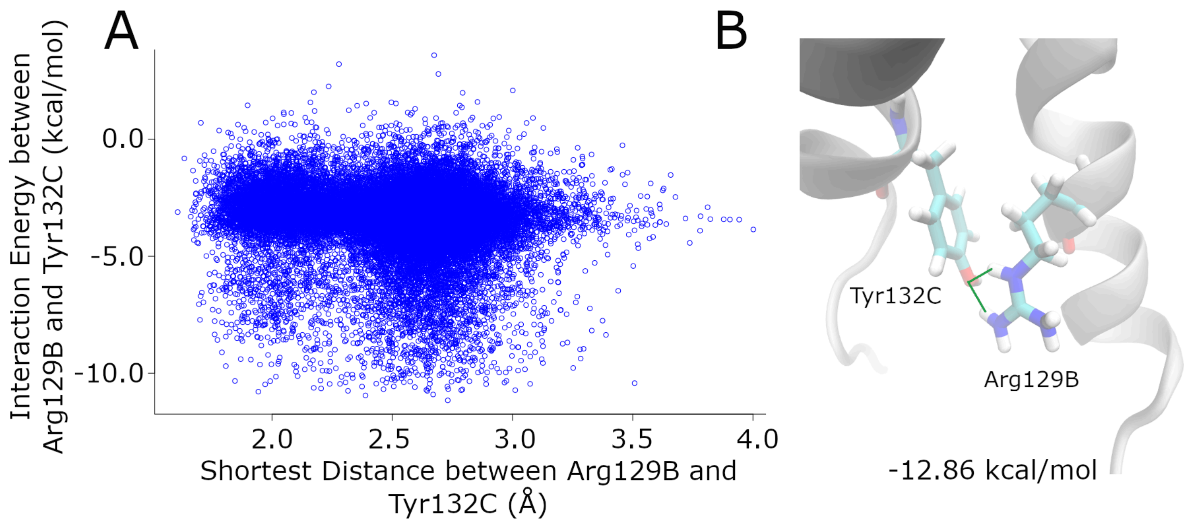

Figure S5: Interaction Arg129B-Arg132C A) Scatter plot of the interaction energy between Arg129B and Arg132C versus the shortest distance between these two residues. B) Atomic representation of the residues Arg129B and Arg132C at their more favorable interaction energy (-12.86kcal/mol) with the hydrogen bonds detected by VMD software drawn in green.

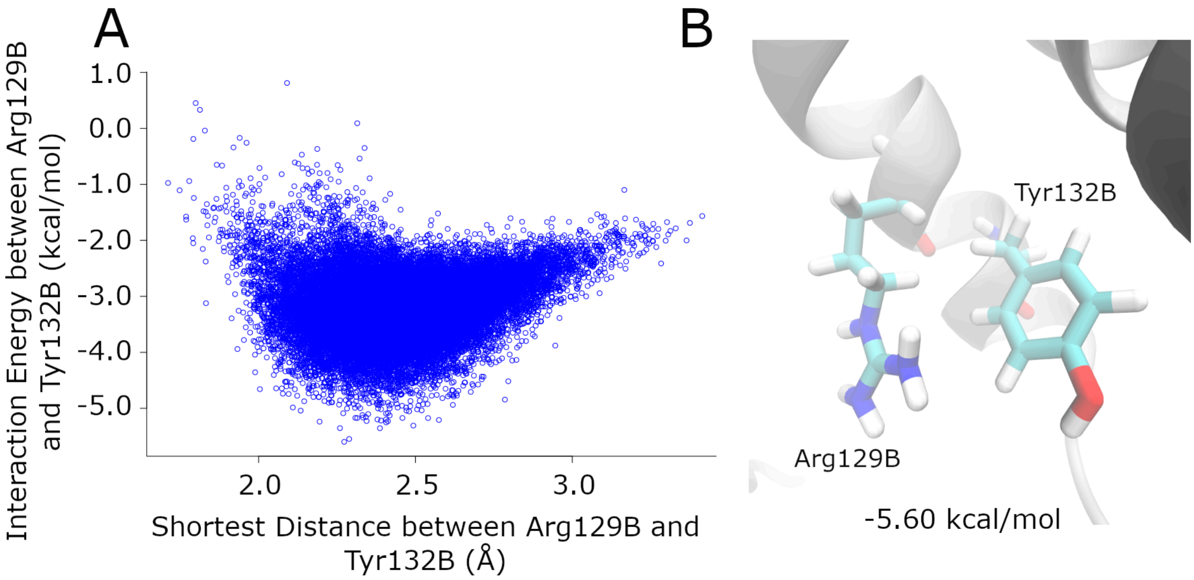

Figure S6: Interaction Arg129B-Arg132B A) Scatter plot of the interaction energy between Arg129B and Arg132B versus the shortest distance between these two residues. B) Atomic representation of the residues Arg129B and Arg132B at their more favourable interaction energy (-5.60 kcal/mol).

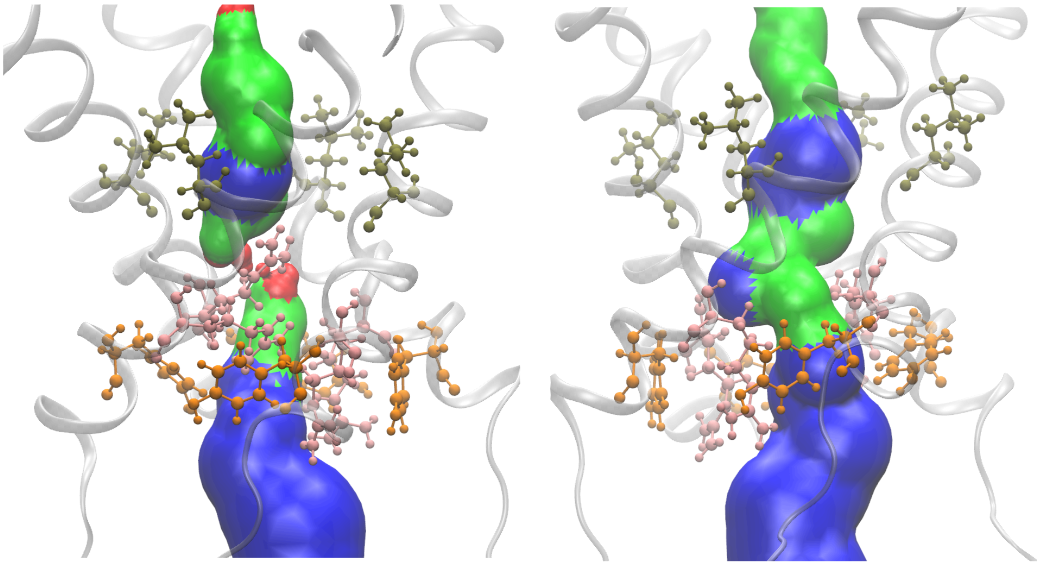

Figure S7: Representation of different states of KirBac3.1 S129R channel.
Left: Representation of the narrowest state **Right:** the widest state. Residues 124 (khaki), 129 (pink), and 132 (orange) are represented. The channels were computed with Hole software. The two frames were taken from the MDeNM relaxed structures. They were selected by comparing the shortest distances between the residues belonging to opposing chains for 124, 132, and 129. (e.g., the distance between the residues 124 chain A and 124 chain C). The smallest distances were found for the left frame and the highest ones in the right frame. In this software the channel diameter is described in three colors related to the number of the water molecule that can go through the channel: zero (red), one (green), two or more (blue)

Parameters	KirBac3.1 WT X-Ray	KirBac3.1 S129R X-Ray	KirBac3.1 S129R closed	KirBac3.1 S129R open
Outer kink chain A (°)	4.41	8.04	10.56	11.58
Outer kink chain B (°)	3.07	7.59	11.52	14.01
Outer kink chain C (°)	4.64	8.09	5.08	7.97
Outer kink chain D (°)	3.79	9.21	11.26	10.44
Pore Radius Leu124 (Å)	1.29	5.44	1.37	2.00
Pore Radius Tyr132 (Å)	0.84	1.86	1.37	2.05
Pore Radius Ser/Arg129 (Å)	2.78	2.26	1.42	2.26

Table S1: Values of the outer TM kink angles and the pore radius at the level of Leu124, Tyr132 and Ser/Arg129 of the crystallographic structures of KirBac3.1 WT (2WLJ), KirBac3.1 S129R (3ZRS), and in representative MDeNM simulated structures of KirBac3.1 S129R in their closed and open conformations. The last two structures are relaxed structure from MDeNM (See Figure S7)
